# Ethical challenges of cord blood banks: a scoping review

**DOI:** 10.25122/jml-2021-0162

**Published:** 2022-06

**Authors:** Madjid Soltani Gerdfaramarzi, Shabnam Bazmi, Mehrzad Kiani, Leila Afshar, Mohsen Fadavi, Seyed Ali Enjoo

**Affiliations:** 1 Department of Medical Ethics, School of Traditional Medicine, Shahid Beheshti University of Medical Sciences, Tehran, Iran

**Keywords:** cord blood bank, ethical challenges, stem cells, thematic content analysis, scoping review

## Abstract

Cord blood is a rich source of hematopoietic stem cells used to treat many diseases of blood origin. Thus, storage banks were created to store and provide umbilical cord cells. With the development of diagnostic and therapeutic technologies and techniques in medicine, ethical issues have also become more widespread and complex. After the creation of the cord blood banks, efforts were made to address the ethical issues associated with such banks. The present study attempts to identify the ethical challenges in these banks in the published studies. Databases including PubMed, Scopus, Web of Science (WOS), Embase, Proquest, and Google Scholar were searched from January 1996 to January 2021. Then, the ethical challenges of the cord blood bank were extracted from the results section using thematic content analysis. 22 studies were selected based on inclusion and exclusion criteria. The ethical challenges raised in the studies included private or public ownership of the bank, fair access to banking services, informed and voluntary consent, failure to provide sufficient information to individuals about the process, confidentiality of user's information, conflict of interest of bank founders (who are commonly doctors). The findings of this study indicated that there are serious ethical concerns regarding umbilical cord blood banks. Responding clearly to these ethical challenges calls for the attention of policymakers and medical ethics professionals; this will require a clear statement of the various aspects of these banks for society.

## INTRODUCTION

Cord blood is a rich source of hematopoietic stem cells widely used to treat blood-borne diseases, including blood cancers, leukemia, thalassemia, congenital anemia, and some defective immune system diseases [1]. Cord blood is the blood remaining in the umbilical cord and placenta after birth and is often discarded, but it is rich in stem cells [2]. These cells can make other types of cells and repair and maintain cells during injury [3]. Thus, if collecting and storing these cells is possible, we are provided with a rich source of hematopoietic stem cells. As mentioned earlier, these cells can be used in blood-related diseases, bone marrow disorders, and immune system dysfunction [4]. Availability, reduced risk of transplant rejection, reduced risk of infection, and the chance of finding a genetically matched specimen are among the most important aspects of cord blood storage [5]. Umbilical cord blood stem cells can only be used in some diseases where the blood cells are not damaged, for a child whose sample has been stored [6]. However, any other person who is genetically compatible Human Leukocyte Antigen (HLA) with the stored blood sample (and the number of cells is appropriate for the patient) can use this blood sample as well [7].

Moreover, cord blood storage banks were established to maintain and supply umbilical cord cells [8]. In Iran, it is estimated that more than 15000 cord blood units have been stored in state-owned banks and more than 150000 units in private banks [9]. Given the increasing demand and need for cord blood cells to perform transplants, the increased number of cord blood banks is reasonable. These banks are currently developing and growing in many countries around the world [10].

Meanwhile, with the development of diagnostic and therapeutic technologies and techniques in medicine, the issue of ethics has become more widespread and complex [11]. In order to use a treatment method, special attention is required to be paid to the ethical standards approved by that given society, especially in medicine [12–15]. With the beginning of the twentieth century, ethics in medical issues entered a new phase, and medical ethics guidelines went beyond the way physicians treated patients. More and more attention must be given to patients' rights in the treatment process, ethical solutions, and lack of secrecy in the treatment process [16]. Issues such as the patient's rights and role in medical decisions, being beneficial, imposing no harm, legal and ethical issues in organ transplantation, justice in resource distribution, informed and willing consent, euthanasia, health and disease, human dignity, ethical issues in fertility and infertility, brain death and its characteristics were included so that no abuse will be made [17].

Ethical considerations are one of the most complex health-related challenges. Health systems attempt to help promote human health by using health methods and processes. Moreover, health care providers in the health sector are required to maintain ethical issues in this sector. Ethical challenges are conflicts and dilemmas raised in the field of ethics. If the activities of health providers are not based on ethical standards, it can lead to unethical behaviors. Thus, serious attention is always required to ethics and related issues concerning health sector issues.

Ethical issues related to cord blood banks also need to be seriously considered by health researchers. After the foundation of these banks, serious efforts were made to review and analyze their related issues. By being aware of the ethical challenges associated with cord blood banks, policymakers can have a better view of the role of ethical issues in such centers and help solve the ethical problems associated with such banks. Thus, the present review study examined the challenges of cord blood banks in the published studies.

## MATERIAL AND METHODS

In this scoping review study, the databases including PubMed, Scopus, Web of Science (WOS), Embase, Proquest, and Google Scholar were searched from January 1996 to January 2021. The search strategy (in Appendix 1) was used to search the PubMed database. This strategy was then adapted to the other databases mentioned above. Moreover, the reference lists of the extracted articles were also searched to find a list of other related studies. The databases were searched independently by two authors; any disagreements between the researchers were resolved through discussion.

### Inclusion criteria

- Studies published in English and released from January 1996 to January 2021;
- Studies published in peer-reviewed journals;
- Studies addressing the ethical challenges of blood banks;
- There were no limitations in terms of geographical location.

### Exclusion criteria

- Studies published in non-English languages;
- Studies that did not address the ethical challenges of blood cell banks in their content and title.

### Data extraction

Two authors independently extracted the initial data of the selected studies. The name of the first author, publication year, geographical location of the study, type of study, and the most important findings of the study were extracted.

### Data analysis

Challenges and problems related to umbilical cord blood banks were summarized in a table. Moreover, thematic content analysis was used to separate the results obtained.

## RESULTS

**Figure 1:**
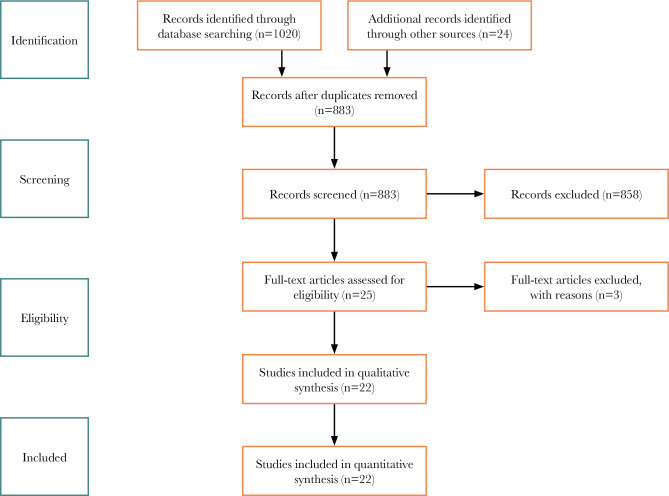
Characteristics of selected studies.

After searching the scientific databases, 1020 articles were found, and 137 duplicate articles were deleted. The titles and abstracts of 883 articles were reviewed, and 858 articles were deleted; they were irrelevant to the subject of the present study. Then, the full texts of the remaining 25 articles were reviewed. Due to irrelevance, another 3 articles were deleted. Finally, 22 articles were selected and analyzed based on inclusion and exclusion criteria [18–39]. Figure 1 illustrates the study selection process.

In investigating the umbilical cord blood bank challenges, based on selected studies, 17 challenges were mentioned. Some of the most important and recurring challenges are introduced in the following sections.

### Private or public ownership of the banks

9 studies investigated private or public ownership of stem cells. Basically, the mechanisms ruling the health sector are not like the other sectors of the economy, and as a result, this sector cannot be completely given to the free market economy. Given the nature of the activities of stem cell banks, whether the managers of these banks were pursuing economic benefits or not was one of the issues mentioned. From reviewing the selected studies, it was concluded that more concerns were about the private or public ownership of stem cell banks, using the services of these banks, and the development of justice in accessing their services. It can be observed that the private sector managers had mostly highlighted the economic issues to increase their capital in these banks; in contrast, the public sector attempted to provide most of the services for those who needed such services.

### Informed consent

In 5 articles, informed consent and related issues were mentioned. Informed consent is one of the most fundamental concepts in medical ethics and patient rights in the world. Acquiring informed and willing consent of the patient before starting any diagnostic and therapeutic activity will lead to positive moral and clinical results. Informed consent, a key component of a patient's rights in stem cell banks, is a process through which the patient or person on his/her behalf understands and agrees with the bank's rules. Informed consent and involving the individuals with the decisions related to themselves will lead to a better understanding of the processes of stem cell banks. The employees of these banks are obliged to provide people with information about all the processes involved.

### Justice in access to services

The nature of justice has always been one of the most important questions in the human mind, and over the years, numerous and varied answers have been provided to this question. Equality is one of the most important criteria and indicators for judging the success of health-related issues. Whenever differences in health outcomes resulted from an irreparable compensable imbalance in the distribution of facilities and access to different social groups or communities, health injustice occurred. In other words, health justice occurs when all members of society can maintain their complete health, and their economic and social status does not affect their health. In relation to stem cell blood banks, 5 studies investigated issues related to justice and the access of people in need of these services. The fact that in such banks, everyone can benefit from these services (regardless of their social and economic conditions) has been a serious challenge.

### Conflict of interests

Three studies addressed the issue of conflict of interest concerning the umbilical cord blood stem cell bank. Conflict of interest is the most important origin of corruption. For this reason, to prevent and control corruption, conflicts of interest must be controlled and managed. However, recognizing and understanding instances of conflict of interest is the first step toward properly managing them. Conflict of interest becomes more important when it occurs in a center or organization that is directly related to the lives and health of people. Conflict of interest refers to a situation in which a person or some people have a credible position and responsibility and are considered trustworthy by the general public, but they have different interests (individual or group) that are in conflict with their position and their own responsibility. In other words, putting a person in a situation where there is a conflict between his/her obligations and his/her own personal interests is called “conflict of interest” in management science. In the health sector, one of the right yet main concerns was to address conflicts of interest in the umbilical cord blood stem cell bank. The main questions include: *"Are the managers of these centers mostly doctors?"* and *"Are all the ongoing processes in these centers conducted regardless of the potential financial and research benefits they might have?"*.

### Social problems

Social perspectives on health issues are of high significance. In order to benefit from appropriate services and develop activities in the health sector, efforts are required to promote people's views about the processes through training and informing people in this regard. Lack of due attention to their social issues is likely to reduce the quality of health services, or it may even prevent people from using such services. Three studies investigated social challenges in the umbilical cord blood stem cell banks. In many societies, there is no positive attitude towards such banks. Individuals' little and sometimes incorrect information has made some of them think that these banks are unnecessary.

### Confidentiality

Confidentiality of activities conducted in the health sector is a fundamental right and cannot be taken away from anyone. Three studies investigated their concerns about the confidentiality of activities in the umbilical cord blood stem cell banks. Reviewing the views of thinkers in the field of privacy law indicates the great complexity of this concept and the difficulties existing in providing a comprehensive definition for “privacy”. Given its nature in different cultures and its definition for different individuals and different ages, and even different times and places, privacy can have its own meaning; this has made it difficult to provide a unique definition. The activities taking place in the umbilical cord blood stem cell banks must be transparent. Many people had doubts about keeping their personal information confidential in such banks. They had their own concerns about what their cells would be used for. The characteristics of the selected studies and their most important results are provided in Table 1.

**Table 1:** Details of the studies included in the review.

First author	Year of publication	Country	Participants	Main finding
Sugarman	1997	United States	Doctors, researchers, people specializing in blood bank, anthropology, bone marrow transplantation, social sciences, women, children and social sciences.	Moral satisfaction of the donor and receiver of cells. Uncertainty of the process of obtaining information. Problems and diseases that may occur after transplantation. Parents' concerns about protecting cells. Private or public ownership of the banks. Problems in the process of obtaining consent.
Samuel	2007	Australia	-	Recipients are aware of the consequences, advantages, and disadvantages of this method Psychological and mental problems caused by receiving cells. Informed and voluntary consent. Insufficient information about processes. Cultural, social and economic problems in society. Economic perspective with increasing private banks and concerns raised against them.
Rao	2012	United States	-	Inappropriate social attitude towards the bank. Parents' obstinacy in accepting a bank for their child's future. Difficulty in diagnosing donor blood disorders. Conflict of interest in establishing a bank (especially private banks).
Hug	2009	Sweden	-	Usage in animal studies. Failure to check the donor in terms of health and the possibility of tracking them. Lack of proper staff training.
Ballen	2010	United States	-	Private or public ownership of the banks
Giacomini	2007	United States	-	Fair access. The recipients little or no information about the consequences, advantages and disadvantages of this method.
Warwick	2010	England	-	Lack of required financial resources. Commercial attitude towards the banks.
Smith	2008	United States	-	Uncertainty about the donors' informed consent. Usage in private or public banks. The way of obtaining individuals' consent or dissatisfaction. Confidentiality of user information. Property rights in banks.
Pinch	2001	United States	-	Conflict of interest of the founders of the banks (being mostly doctors). Individuals' consent. Tracking donors. Privacy and confidentiality.
Petrini	2013	Italy	-	Respect for human dignity. Lack of commercialization. Justice in access to services. Respect for privacy.
Turkmen	2008	Turkey	-	Avoiding harm to individuals receiving cells. Respect for personal independence and justice.
Childress	2004	United States	-	Possibility of cloning. Financial issues.
Lucea	2012	Spain	-	Is its clinical application morally right or not? Are financial issues influential? The difference between being private or public. Lack of moral support for associations and groups Problems between parents for cell transfer.
Serrano-Delgado	2009	Mexico	-	Lack of proper definition of banks and raising concerns. Confidentiality of donor's information. Informed consent. Private or public ownership of the banks.
Chima	2011	South Africa	-	Available only in the private sector. Consent and ownership of tissues. Cost-effectiveness of cell harvesting. Unfairly limited to the rich only.
Petrinin	2010	Italy	-	Informed ownership satisfaction. Frameworks. Public Banks vs. Commercial Banks. Ability to track advertising costs. Commercialization. Relationships between patients, doctors, and cord blood banks. Relationships between recipients and cord blood banks.
Mendes-Takao	2010	Brazil	-	Issue of property rights (donor child or his/her parents). Principles of Justice.
Alahmad	2020	Saudi Arabia	Qualitative study	Usage limits of the cell. Informed consent. Ethical rules and regulations.
Kin	2014	United States	-	Imposing pressure on consent issues. Justice. Necessities to minimize the risks of injury. Informed consent.
Abberton	2019	Australia	-	Acquiring consent. Ethical approval of studies and activities conducted by the banks.
Weisbrot	2012	Australia	-	Privacy. Avoiding discrimination and establishing justice in access. Informed consent.
Salvaterra	2006	Italy	-	The supervision of ethics committed over the activities conducted by the banks. Private or public ownership of the banks.

## DISCUSSION

The present study was conducted to identify the ethical challenges of cord blood banks. The increasing development of human knowledge, the promotion of biomedical technologies, and the increased capabilities of researchers in diagnosing and treating various diseases have been accompanied by many challenging issues, especially the ethical ones [40]. Despite advances in medical technology, there are concerns about the ethical issues associated with them. These concerns led to the emergence of movements on the theme of patients' rights as well as the right of society to participate in medical decision-making [41]. Addressing ethical issues related to these technologies seems to be one of the basic requirements that must be considered in the comprehensive health system [42]. One of these issues is respect for the autonomy and independence of individuals (sick or healthy) who are entitled to choose/reject a special treatment [43]. The maximum benefit for the recipients of health services should be considered by the service providers. Moreover, there should always be no harm to people. Finally, people are required to use health services regardless of their social and economic status; justice must be provided to achieve those services [44].

In our selected studies, various issues were raised as challenges to cord blood banks. One of the challenges mentioned in some studies was answering: *"Should cord blood banks be private or public?"*. In a private cord blood bank, the sample storage is conducted after the request of the applicant and after paying the related costs. The blood sample is owned by the contracting party [20–22]. In a general cord blood bank, a sample of cord blood is donated to the bank by the family, the storage is thus free, and the sample is owned by the general cord blood bank. The most important issue in this regard is providing justice in access to bank services [22]. Ethically speaking, people should be able to easily use the bank services if needed. However, in practice, people cannot keep their samples with private banks for various reasons, including lack of suitable economic status. Thus, they use the services of public banks, and they may be on the waiting list for a long time to receive services. Such conditions may even endanger their lives [45].

Studies have indicated that people who do not have adequate financial and insurance conditions are at a higher risk of illness and prefer to spend their money on housing, food, and living expenses [46]. Justice in accessing health services for these people has diminished and is at odds with ethical issues. However, one of the goals of founding private cord blood banks is to provide easier conditions for people with better financial status [47]. Health policymakers must do their best to provide a platform for the general public. In this way, a platform is created for people who want to use faster services at their own expense. Moreover, this platform allows the public to use the services provided by cord blood banks in better conditions.

One of the challenges raised in the selected studies was the informed consent of individuals to donate or store umbilical cord blood [26]. Acquiring consent and informing individuals about health-related issues is not new to treatment or diagnostic procedures and has been done for a long time [32]. Nowadays, given the increasing complexity of the nature of medical activities, it is necessary to inform the patients about the processes that the health sector performs for him/her. Failure to inform individuals can be a major obstacle to conducting the required medical activities [11, 12, 16]. As for umbilical cord blood, individuals must be aware of the steps of receiving and using them for themselves or others and grant their complete consent for conducting such activities [34]. In other words, one of the most important issues in the field of medical ethics is the need for patients' consent to perform the activities conducted by cord blood banks. Obtaining such consent before the activities conducted by these banks will result in positive moral and clinical results [38].

Another challenge raised in the selected reviewed studies is disclosing the names of donors and recipients of services or keeping them confidential, especially in public cord blood banks. Service providers in these banks must strive to keep the names of the beneficiaries confidential. However, some raise the very question of *"Can the cord blood donor be informed of the identity of the recipient?"* [36]. Protecting the information of individuals in these banks is of high significance, and attempts to disclose or keep the information confidential based on the donor's request led to numerous discussions between proponents and opponents [29, 30].

Problems and illnesses that are likely to develop in recipients after using the cord blood of others were other ethical challenges mentioned in the studies. Most individuals may be at risk of disease when receiving an organ from someone else [19]. The nature of umbilical cord blood can cause changes and create a background for potential diseases for the recipients. Changes are likely to be made in the recipient's health owing to receiving cord blood; whether or not they become ill is a serious concern in such individuals [32–36]. Researchers and providers of cord blood services are in morally difficult conditions. It is not possible for researchers and umbilical cord blood providers to diagnose many blood disorders in the donor. They attempt to provide and transplant the best cord blood by knowing the donor's physical conditions and medical record [17]. The role of ethics in diseases that may be caused by umbilical cord blood transplants has created serious ethical challenges among researchers in this field. Who is responsible if the recipients get sick? As previous studies stated, avoiding patient harm is an ethical principle [48, 49]. However, the fact is that service providers may have little knowledge about the damage caused by cord blood. Moreover, people may have serious emergency needs to use cord blood; this raises serious ethical concerns [50].

Another challenge reviewed in studies of umbilical cord blood is the extent and limits of using these cells. How much of these cells can really be used? The serious moral concern raised in this regard is “cloning”. Besides bringing many social and personal fruits to human beings, scientific studies also caused many moral problems [51]. Serious concerns about using cord blood as an example of “cloning” have been raised in many scientific societies. In this regard, various studies referred to the lack of adequate supervision over the processes ruling the banks [30]. Many opponents linked using cord blood with religious issues; they believe using these cells contradicts religious themes. Moreover, the opponents believe that using these cells should be very limited, and developing their usage is required to be under strict supervision and rules [52].

Another serious challenge with cord blood banks is that different countries have their own ethical rules and regulations for their usage. Different studies referred to the lack of adequate supervision for ethical issues in the banks [29]. The fact that some individuals ignore ethical issues for their own financial and economic interests is a serious challenge. Ethical rules and guidelines are highly valuable in answering this question: *"How should appropriate measures be taken in a particular situation?"* [36]. When these rules do not exist or are not implemented properly, there might be a moral challenge or crisis. Serious evidence can be found about the lack of ethical guidelines in medicine in many countries of the world; strict moral laws must be applied with strict supervision over cord blood banks [44].

Another challenge mentioned in the studies was parents' concerns about protecting their children's cells in banks. Parents had serious concerns about using their children's cells in experiments and using them without permission for other people. Different studies indicate that umbilical cord blood cells should not be used without parents' permission. This moral concern is right and reasonable; cord blood bank managers are required to give parents serious moral and legal guarantees about this concern [42].

Many managers of cord blood banks are doctors. Given the scope of their activities in health, the issue of conflict of interests was also raised as one of the challenges of these banks. In the reviewed studies, the idea of making more financial benefit in private banks and using medical tests without obtaining permission from the owner of the umbilical cord blood are highly emphasized. Given its high significance, the health domain is exposed to a high prevalence of conflict of interests manifested in different forms [36–38]. This conflict of interests sometimes manifests as interest (financial or non-financial) between members of the medical team and the community itself. Legal and ethical mechanisms are required to monitor the activities of banks. Moreover, there are serious concerns about whether the managers of these centers should be doctors or non-doctors [12].

This study had some limitations, some of which can be mentioned. We only reviewed studies published in English, and studies published in non-English languages could have provided newer findings on the challenge of cord blood banks. Moreover, the design of selected studies can affect the findings as well.

## CONCLUSION

The findings of this study showed that the existence of cord blood banks in different countries is accompanied by serious ethical challenges and concerns. Using cord blood as a scientific method for many diseases has been absorbing and fascinating by scientists and researchers. Many countries established umbilical cord blood banks. In the meantime, given the significance of health activities and their role in the individuals' quality of life and human dignity, ethical issues and concerns related to medical activities (either in the field of prevention, diagnosis or treatment) are required to be taken into account. Thus, before creating cord blood banks, health policymakers need to consider the ethical problems and challenges and do their best to solve such problems and challenges.

## Appendix

**Appendix 1:** Search strategy and number of results for each database.

Database	Search strategy	Results
Embase	((“Stem Cell Transplantation”:ti,ab AND “Placental Blood”:ti,ab) OR “Umbilical Cord Blood Stem Cell Transplantation”:ti,ab OR “Placental Blood Stem Cell Transplantation”:ti,ab OR (“Stem Cell Transplantation”:ti,ab AND “Cord Blood”:ti,ab) OR (“Blood Stem Cell Transplantation”:ti,ab AND “Umbilical Cord”:ti,ab) OR “umbilical cord blood stem cell”:ti,ab OR “umbilical cord blood stem cell bank”:ti,ab OR “cord blood stem cell bank”:ti,ab OR “cord blood stem cell transplantation”:ti,ab OR “cord blood bank”:ti,ab OR “public private hybrid cord blood bank”:ti,ab) AND (“Ethical challenge”:ti,ab OR “ethical challenges”:ti,ab OR “ethical issue”:ti,ab OR “ethical issues”:ti,ab OR “Ethics Code”:ti,ab OR “Ethics Codes”:ti,ab OR “Code of Ethics”:ti,ab OR “Ethical Codes”:ti,ab OR “Ethical Code”:ti,ab OR “Ethical Directives”:ti,ab OR “Ethical Directive”:ti,ab OR “Codes of Professional Ethics”:ti,ab OR “Professional Ethics Code”:ti,ab OR “Professional Ethics Codes”:ti,ab OR ethic/exp OR “ethical aspects of cord blood”:ti,ab OR “ethical challenges of cord blood bank”:ti,ab OR “ethic* problem*”:ti,ab OR “ethic* consider*”:ti,ab)	19
Scopus	((TITLE-ABS-KEY("Stem Cell Transplantation") AND TITLE-ABS-KEY("Placental Blood")) OR TITLE-ABS-KEY("Umbilical Cord Blood Stem Cell Transplantation") OR TITLE-ABS-KEY("Placental Blood Stem Cell Transplantation") OR (TITLE-ABS-KEY("Stem Cell Transplantation") AND TITLE-ABS-KEY("Cord Blood")) OR (TITLE-ABS-KEY("Blood Stem Cell Transplantation") AND TITLE-ABS-KEY("Umbilical Cord")) OR TITLE-ABS-KEY("umbilical cord blood stem cell") OR TITLE-ABS-KEY("umbilical cord blood stem cell bank") OR TITLE-ABS-KEY("cord blood stem cell bank") OR TITLE-ABS-KEY("cord blood stem cell transplantation") OR TITLE-ABS-KEY("cord blood bank") OR TITLE-ABS-KEY("public private hybrid cord blood bank")) AND (TITLE-ABS-KEY("Ethical challenge") OR TITLE-ABS-KEY("ethical challenges") OR TITLE-ABS-KEY("ethical issue") OR TITLE-ABS-KEY("ethical issues") OR TITLE-ABS-KEY("Ethics Code") OR TITLE-ABS-KEY("Ethics Codes") OR TITLE-ABS-KEY("Code of Ethics") OR TITLE-ABS-KEY("Ethical Codes") OR TITLE-ABS-KEY("Ethical Code") OR TITLE-ABS-KEY("Ethical Directives") OR TITLE-ABS-KEY("Ethical Directive") OR TITLE-ABS-KEY("Codes of Professional Ethics") OR TITLE-ABS-KEY("Professional Ethics Code") OR TITLE-ABS-KEY("Professional Ethics Codes") OR ALL(ethic*) OR TITLE-ABS-KEY("ethical aspects of cord blood") OR TITLE-ABS-KEY("ethical challenges of cord blood bank") OR TITLE-ABS-KEY("ethic* problem*") OR TITLE-ABS-KEY("ethic* consider*"))	744
Web of Science	((TS=("Stem Cell Transplantation") AND TS=("Placental Blood")) OR TS=("Umbilical Cord Blood Stem Cell Transplantation") OR TS=("Placental Blood Stem Cell Transplantation") OR (TS=("Stem Cell Transplantation") AND TS=("Cord Blood")) OR (TS=("Blood Stem Cell Transplantation") AND TS=("Umbilical Cord")) OR TS=("umbilical cord blood stem cell") OR TS=("umbilical cord blood stem cell bank") OR TS=("cord blood stem cell bank") OR TS=("cord blood stem cell transplantation") OR TS=("cord blood bank") OR TS=("public private hybrid cord blood bank")) AND (TS=("Ethical challenge") OR TS=("ethical challenges") OR TS=("ethical issue") OR TS=("ethical issues") OR TS=("Ethics Code") OR TS=("Ethics Codes") OR TS=("Code of Ethics") OR TS=("Ethical Codes") OR TS=("Ethical Code") OR TS=("Ethical Directives") OR TS=("Ethical Directive") OR TS=("Codes of Professional Ethics") OR TS=("Professional Ethics Code") OR TS=("Professional Ethics Codes") OR ALL=(ethic*) OR TS=("ethical aspects of cord blood") OR TS=("ethical challenges of cord blood bank") OR TS=("ethic* problem*") OR TS=("ethic* consider*"))	57
Proquest	((“Stem Cell Transplantation” AND “Placental Blood”) OR “Umbilical Cord Blood Stem Cell Transplantation” OR “Placental Blood Stem Cell Transplantation” OR (“Stem Cell Transplantation” AND "Cord Blood") OR ("Blood Stem Cell Transplantation" AND "Umbilical Cord") OR "umbilical cord blood stem cell" OR "umbilical cord blood stem cell bank" OR "cord blood stem cell bank" OR "cord blood stem cell transplantation" OR "cord blood bank" OR "public private hybrid cord blood bank") AND TI,AB("Ethical challenge" OR "ethical challenges" OR "ethical issue" OR "ethical issues" OR "Ethics Code" OR "Ethics Codes" OR "Code of Ethics" OR "Ethical Codes" OR "Ethical Code" OR "Ethical Directives" OR "Ethical Directive" OR "Codes of Professional Ethics" OR "Professional Ethics Code" OR "Professional Ethics Codes" OR ethic* OR "ethical aspects of cord blood" OR "ethical challenges of cord blood bank" OR "ethic* problem*" OR "ethic* consider*")	117
